# Stressful environments favor deceptive alternative mating tactics to become dominant

**DOI:** 10.1186/s12915-023-01664-5

**Published:** 2023-07-27

**Authors:** Maria J. Albo, Camila Pavón-Peláez, Mauro Martínez Villar, Bruno A. Buzatto, Ivanna Tomasco

**Affiliations:** 1 Sección Entomología, Facultad de Ciencias, Universidad de La República, Montevideo, Uruguay; 2 Departamento de Ecología y Biología Evolutiva, Instituto de Investigaciones Biológicas Clemente Estable, Montevideo, Uruguay; 3 Programa de Desarrollo de las Ciencias Básicas (PEDECIBA), Universidad de La República, Montevideo, Uruguay; 4 Departamento de Ecología y Evolución, Facultad de Ciencias, Universidad de La República, Montevideo, Uruguay; 5 Flinders University, Adelaide, Australia; 6 Macquarie University, Sydney, Australia

**Keywords:** Evolutionary stable strategies, Gift-giving spiders, Worthless gifts

## Abstract

**Background:**

Deceptive alternative mating tactics are commonly maintained at low frequencies within populations because males using them are less competitive and acquire lower fitness than those using dominant tactics. However, the successful invasion of a male deceptive tactic is plausible if deception carries no fitness cost to females. Among populations of the gift-giving spider *Paratrechalea ornata*, males very often offer females a deceptive worthless gift, rather than a nutritive gift*.* We tested the degree to which deceptive worthless gifts can occur in natural populations living under divergent environmental conditions (moderate and stressful). We examined the plasticity of morphological and behavioral traits and analyzed the fitness of females in relation to the gift type, also examining the paternity acquired by males offering either gift type.

**Results:**

We demonstrated that worthless gifts can become dominant under highly stressful environmental conditions (84–100%). Individuals in such environment reach smaller sizes than those in moderate conditions. We suggest that the size reduction probably favors low metabolic demands in both sexes and may reduce the costs associated with receiving deceptive worthless gifts for females. In contrast, males living under moderate conditions varied the use of the deceptive tactic (0–95%), and worthless gifts negatively influenced female fecundity. Furthermore, male size, rather than gift content, positively impacted paternity success in the moderate but not in the stressful environment.

**Conclusions:**

Overall, this is the first empirical evidence that a reversible deceptive tactic can become dominant when the environment becomes harsh and mate choice becomes limited.

**Supplementary Information:**

The online version contains supplementary material available at 10.1186/s12915-023-01664-5.

## Background

Trait evolution is affected by the invasion of new mutants, and increased variation in the fitness of dominant phenotypes creates the opportunity for alternative phenotypes to spread in the populations [1]. The evolutionary dynamics of new alternative phenotypes replacing the original ones has been theoretically [2–6] and empirically shown for conditional irreversible phenotypes [7, 8], but never for deceptive and reversible phenotypes in alternative mating tactics. This is unsurprising, as the opportunistic nature of these tactics creates unpredictability of fitness effects and relative frequencies. Importantly, deception is costly for receivers, in this case females [9, 10], and thus, a large-scale spread of these deceptive tactics is usually limited by the low fitness benefits associated with them [5, 11–13]. Likewise, most alternative mating tactics represent a conditional strategy, where a dominant tactic is used by the majority of individuals, whereas the deceptive tactic remains rare due to frequency-dependent selection [11, 14–16].

In conditional strategies, each individual can express different tactics, depending on their environment and/or own condition [11]. Typically, the largest, territorial, or dominant males have the highest fitness, whereas those unable to reach such standards acquire matings through alternative ways, such as sneaking or using satellite tactics [5, 11–13]. This is the case in some nuptial gift-giving species, where males offering deceptive worthless gifts increase their mating success over those without gifts but achieve lower fitness than those offering nutritive gifts [17–19]. For example, males from the dance fly *Rhamphomya sulcata* offer nutritive gifts to females during courtship [17]. During mating, females feed from the gift, and thus, sperm transfer is dependent on the gift size. If males offering non-nutritive “worthless” gifts are introduced, females would accept them but mate for a shorter time than when mating with males offering large nutritive gifts [17]. Similarly, in the spider *Pisaura mirabilis*, females limit sperm transfer when the gift is worthless, and therefore, the deceptive tactic is maintained at low frequencies in the field [18]. Sensory exploitation of female foraging motivation has been a central hypothesis for the origin of nuptial gifts [20]. Commonly, in these mating systems, females gain food and increase their fecundity by accepting multiple matings and food gifts [21]. The successful spread of a deceptive tactic such as worthless gifts is plausible, however, if females are unable to detect deception or are indifferent to gift content [9]. In such cases, mating success would depend on other male attributes rather than gift type, and males using the deceptive tactic can gain equal or higher fitness than those using the dominant tactic. In a scenario in which all males gain similar reproductive success regardless of the gift type, offering a nutritive gift (i.e., performing the initially dominant mating tactic) becomes costly for males due to reducing food intake without added fitness benefits, and this tactic is expected to be replaced over evolutionary time.

Here, we investigated this process in the gift-giving spider *Paratrechalea ornata*, in which individual males can vary the gift content, by either wrapping an insect prey in silk (nutritive gift) or wrapping inedible items, such as prey leftovers or plant parts (worthless gift) gathered from the surroundings [22–24]. In some populations, the frequency of worthless gifts is low (average 40%), whereas in others, it is very high (average 70%) [23], providing an ideal scenario for studying how deceptive mating tactics can become widespread. Firstly, individuals live in unpredictable and stressful environments that limit their life cycle and survival (i.e., limited prey availability) [25–29]. Therefore, males are under strong selective pressures to gather enough resources for survival, while they also need to allocate prey to produce nuptial gifts. Stressful environmental conditions would restrict the opportunity for males to develop their main sexually selected trait (nutritive nuptial gifts wrapped in silk), as well as other traits [27, 30–33]. Secondly, studies in a population with a relatively low frequency of worthless gifts have shown males respond to limited prey availability by adding extra silk to their worthless gifts [24]. Adding more silk slightly increases the costs of producing deceptive gifts, but with the benefit of gaining similar mating success to when offering nutritive gifts [22, 23, 34, 35]. This elevated mating success of offering worthless gifts is probably because silk wrapping of the gift can also act as a signal for mate attraction [35, 36] and because females would only recognize the gift content once they accept the mating and start to feed on the gift, as has been suggested for other spiders [18]. Opposite to expectations, in this *P. ornata* population, mating duration can be similar for both gift types when mating with unmated females [22, 23, 34], though recent findings have revealed that mating duration with worthless gifts can be reduced under sperm competition [37]. Additionally, other traits such as male size positively correlate with mating duration [38]. Finally, there is no clear indication that cryptic female choice for gift content can play a role after mating [34]. We hypothesize that in highly stressed populations, there is a lack of female choice for gift type, creating the opportunity for the unlimited spread of deceptive worthless gifts. This switch in the frequencies of male mating tactics in the populations can be possible when female fitness costs (i.e., fecundity) associated with receiving worthless gifts are absent, and there is no correlation between males’ paternity success and gift content.

We used three approaches to demonstrate the potential for a large spread of deceptive worthless nuptial gifts (Additional file: Fig. S1). We first studied two natural populations with moderate and highly stressful conditions for organisms (i.e., climatic variation). We showed that when the environment has permanent prey availability limitations, individuals are smaller and the frequency of worthless gifts reaches almost 100%. In contrast, when the environment is less stressful and has occasional prey limitations, individuals can grow larger and males more often produced nutritive gifts. Secondly, we raised individuals from both populations under high and low prey availability and verified the morphological and behavioral constraints previously found in the field. That is, individuals are the smallest and commonly produce worthless gifts in the highly stressful compared to the less stressful environment. Finally, we exposed females to males offering nutritive or worthless gifts in both populations. Only in the permanent prey-limited environment did females receiving either gift type acquired similar fitness. Gift type did not determine paternity success in either population, whereas females favored large males in the less stressful environment. Our data demonstrate that deceptive gift-giving can be a high-fitness tactic that can become dominant, particularly in stressful environments.

## Results

### Worthless gifts in natural populations: ecological and individual effects

**Fig. 1:**
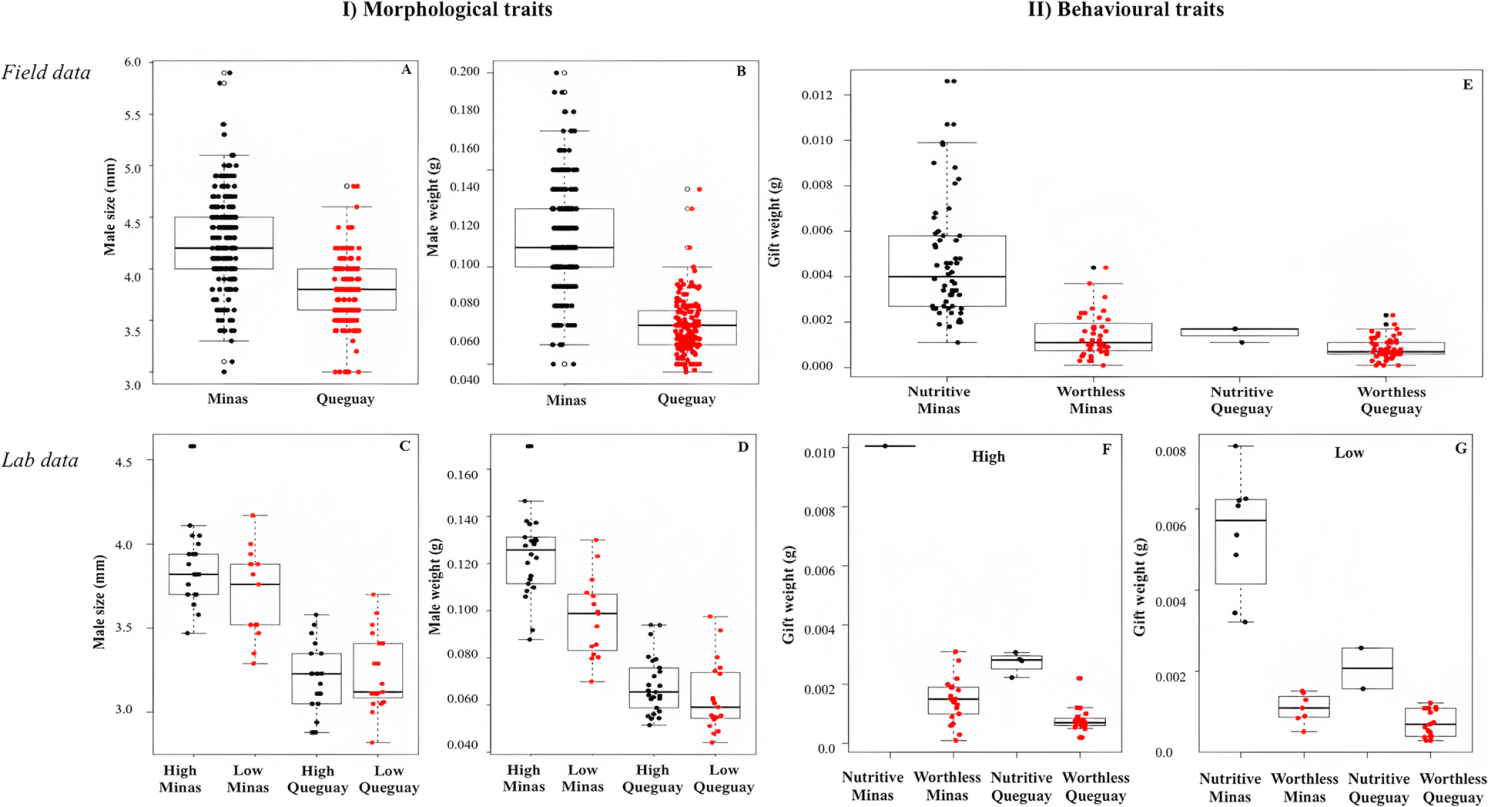
Males morphological and behavioral traits. Individual size, weight, and nuptial gifts obtained from the field and experimental trials performed after 12 weeks of feeding treatment using the two studied *Paratrechalea ornata* populations (Minas and Queguay). (I) Field data showing male size (**A**) and weight (**B**) (*n*_Minas_ = 224, *n*_Queguay_ = 164) and laboratory data showing the effects of prey availability (high and low) on male size (*C*) and weight (*D*) (*n*_High Minas_ = 22, *n*_Low Minas_ = 15, *n*_High Queguay_ = 25, *n*_Low Queguay_ = 19). (II) Field data showing nutritive and worthless nuptial gifts weight (**E**) (*n*_Minas Nutritive_ = 59, *n*_Minas Worthless_ = 40, *n*_Queguay Nutritive_ = 3, *n*_Queguay Worthless_ = 49) and laboratory data showing the effects of high (**F**) and low (**G**) prey availability on nuptial gift weight (*n*_High Minas_ = 22, *n*_Low Minas_ = 15, *n*_High Queguay_ = 25, *n*_Low Queguay_ = 19). In the boxplot, the black line represents the median; gray dots represent outliers. In (I), black dots represent the data points from Minas and red from Queguay; in (II), black dots represent the data points of nutritive and red of worthless gifts. Detailed data information can be found at the “Availability of data and materials” section

We examined the variations in relation to individuals’ size and weight, the frequency of deceptive worthless gifts and prey availability in the two natural populations. The data showed substantial differences between populations related to the males’ morphological and behavioral traits. Male size (estimate =  − 0.51, SE = 0.04, *p* < 0.0001) and weight (estimate =  − 0.05, SE = 0.002, *p* < 0.0001) were lower for Queguay (Fig. 1AB); the same occurred for the females’ morphological traits (size and weight) (Additional file: Table S1). The proportion of males carrying a worthless gift (prey leftovers or plant parts) was lower for Minas (38%) compared to Queguay (96%) (Additional file: Table S1). Worthless gifts were significantly lighter than nutritive gifts (worthless or nutritive; estimate =  − 1.30, SE = 0.13, *p* < 0.0001) and differed between populations (Minas or Queguay; estimate =  − 1.02, SE = 0.38, *p* = 0.01), with no significant interaction term (estimate = 0.55, SE = 0.41, *p* = 0.18; Additional file: Table S1; Fig. 1E). Nutritive gifts from Minas were heavier than all other gifts, while worthless gifts from this population were heavier than worthless gifts from Queguay. We found that absolute prey number and prey number per spider were approximately 6 × higher in Minas and with a broader variation compared to Queguay (Additional file: Table S1).

Considering ecological and individual effects on the probability of producing worthless gifts, our model selection resulted in two most parsimonious models with an AIC_*c*_ difference < 2 (Table 1). Both models (and the top nine models) included an effect of population (from the top model: estimate = 3.30, SE = 0.64, *p* < 0.0001) on the worthless gift proportion, and the relative importance of this variable (sum of AIC weights of all models including the variable) was 98.3%. Additionally, the second-best model showed a negative effect of prey availability (estimate =  − 0.36, SE = 0.17; *p* = 0.03) on the proportion of worthless gifts with a relative importance of 25%.

**Table 1:** Worthless gifts in natural populations: ecological and individual effects. Ranked (based on AIC_*c*_) candidate models with combinations of the fixed effects of ecological (prey number = “Prey”, population = “Pop”) and individual (males and female sizes respectively “Mal size” and “Fem size”) variables on the probability of males producing worthless gifts. All models included the dates generated from a combination of month and year (2015 and 2016) as random effects. Significant *p* values are in bold. Data in bold indicate the two simplest and most parsimonious models

No	Fixed effects	*k*	AIC*c*	ΔAIC_*c*_	Weight	Log- likelihoods	Cumulative weight
**1**	**Pop**	**3**	**45.164**	**–**	**0.4848**	** − 18.0819**	**0.485**
**2**	**Pop + Prey**	**4**	**46.446**	**1.282**	**0.2554**	** − 16.3657**	**0.740**
3	Pop + Fem size	4	48.053	2.889	0.1143	− 17.1694	0.854
4	Pop + Mal size	4	49.848	4.684	0.0466	− 18.0669	0.901
5	Pop + Mal size + Fem size	5	50.345	5.181	0.0363	− 15.1725	0.937
6	Pop + Prey + Mal size	5	52.329	7.166	0.0135	− 16.1647	0.951
7	Pop + Prey + (Pop × Prey)	5	52.594	7.430	0.0118	− 16.2969	0.963
8	Pop + Prey + Fem size	5	52.648	7.484	0.0115	− 16.3240	0.974
9	Pop + Fem size + . (Pop × Fem size)	5	53.978	8.814	0.0059	− 16.9891	0.980
10	Mal size + Fem size + (Mal size × Fem size)	5	53.994	8.830	0.0059	− 16.9968	0.986
11	Prey + Fem size	4	54.392	9.228	0.0048	− 20.3390	0.991
12	Prey + Mal size	4	55.501	10.337	0.0028	− 20.8932	0.994
13	Pop + Mal size + (Pop × Mal size)	5	55.732	10.568	0.0025	− 17.8660	0.996
14	Fem size	3	56.675	11.512	0.0015	− 23.8377	0.998
15	Prey + Mal size + (Prey × Mal size)	5	57.970	12.806	0.0008	− 18.9851	0.998
16	Prey + Fem size + (Prey × Fem size)	5	58.394	13.231	0.0006	− 19.1972	0.999
17	Pop + Prey + Mal size + Fem size	6	58.801	13.637	0.0005	− 15.0006	1.000
18	Prey + Mal size + Fem size	5	60.437	15.273	0.0002	− 20.2185	1.000
19	Mal size + Fem size	4	60.519	15.355	0.0002	− 23.4025	1.000
20	Mal size	3	64.442	19.278	0.0000	− 27.7208	1.000
21	Prey	3	75.821	30.657	0.0000	− 33.4106	1.000
22	*Null*	2	90.633	45.469	0.0000	− 42.6499	1.000

### Common garden experiment: high and low prey availability

In this experiment, we raised individual males under high and low prey availability and explored the same morphological (size and weight) and behavioral (frequency of worthless gifts) traits, confirming the observed patterns found in the field. After the feeding treatment (*high* and *low prey availability*), males from Minas were larger and heavier than males from Queguay. The feeding treatment was effective, and the final male size and weight differed between high and low prey availability. The significant interaction term indicated that the feeding treatment had a stronger effect on males in Minas than in Queguay (Additional file: Table S2; Fig. 1CD). Females were raised under high prey availability, and as expected, their size and weight did not differ between feeding treatments but between populations (Additional file: Table S2).

We found a high proportion of worthless gifts produced by males from both populations (Additional file: Table S3). There was a significant interaction between population and feeding treatment on worthless gift production (Table 2). In High-Minas, most males (95%) offered a worthless gift to females during courtship (18 exuviae and 3 houseflies leftovers). In contrast, 60% offered worthless gifts in Low-Minas (3 exuviae and 6 houseflies leftovers). Meanwhile, males from Queguay produced between 84% worthless gifts (15 exuviae and 6 houseflies leftovers) in the high and 89% (6 exuviae and 11 houseflies leftovers) in the low feeding treatment, respectively. Gift weight was different between feeding treatments with a significant interaction with the population. Nutritive gifts were heavier than worthless gifts, and this effect was larger in the high-feeding treatment and in Minas (Table 2, Fig. 1FG).

**Table 2:** Common garden experiment: high and low prey availability. Results from the generalized lineal models (GLMs) with response variables: worthless gift proportion, gift weight (g), silk wrapping duration (total time that males spend wrapping the gift in min), number of silk wrapping bouts (the sum of the total number of wrappings), latency of gift offering (time in min from when the male finished wrapping the gift until he offered it to female), and female acceptance (occurrence of females grabbing gift) in relation to gift type (nutritive or worthless), population, feeding treatment, and the interaction of both population and feeding treatment. Note that gift type was not included in the model when examining worthless gift proportion and gift weight. Significant *p* values are shown in bold

	**Worthless gift proportion**			**Gift weight**			**Silk wrapping duration**			**N silk wrapping bouts**			**Latency of gift offering**			**Female acceptance**		
	**Estimate**	**SE**	***P***	**Estimate**	**SE**	***P***	**Estimate**	**SE**	***P***	**Estimate**	**SE**	***P***	**Estimate**	**SE**	***P***	**Estimate**	**SE**	***P***
Population	− 1.39	1.16	0.23	− 0.43	0.23	0.06	0.67	0.58	0.25	− 0.05	0.18	0.79	0.05	0.37	0.88	17.88	3391.4	0.99
Feeding treatment	− 2.64	1.15	**0.02**	0.62	0.26	**0.02**	− 0.83	0.72	0.26	− 0.18	0.25	0.49	0.33	0.46	0.48	0.52	1.75	0.75
Population × feeding treatment	3.12	1.48	**0.03**	− 0.76	0.35	**0.03**	− 0.86	0.96	0.37	0.02	0.33	0.94	0.60	0.61	0.33	− 19.75	3391.4	0.99
Gift type	–	–	–	–	–	–	2.41	0.65	**0.001**	0.68	0.28	**0.02**	− 0.02	0.41	0.96	1.87	1.21	0.12

Regardless of the feeding treatment, males that produced worthless gifts largely invested in silk deposition in both Minas and Queguay. They spent more time and added more silk wrapping bouts to the exuviae or housefly leftovers than those wrapping a nutritive prey gift. We found no differences between populations and feeding treatment for the other behavioral traits (gift offering and female acceptance) (Table 2, Additional file: Table S3).

### Mating, fecundity, and paternity success of males offering worthless gifts

Here, we analyzed the effect of male gift type (nutritive or worthless) on mating traits (mating duration and number of pedipalp insertions) and fitness traits (fecundity, number of spiderlings, and hatching success) and investigated whether gift type affects a male’s paternity share when females mate multiply.

#### Mating and fecundity traits

**Fig. 2:**
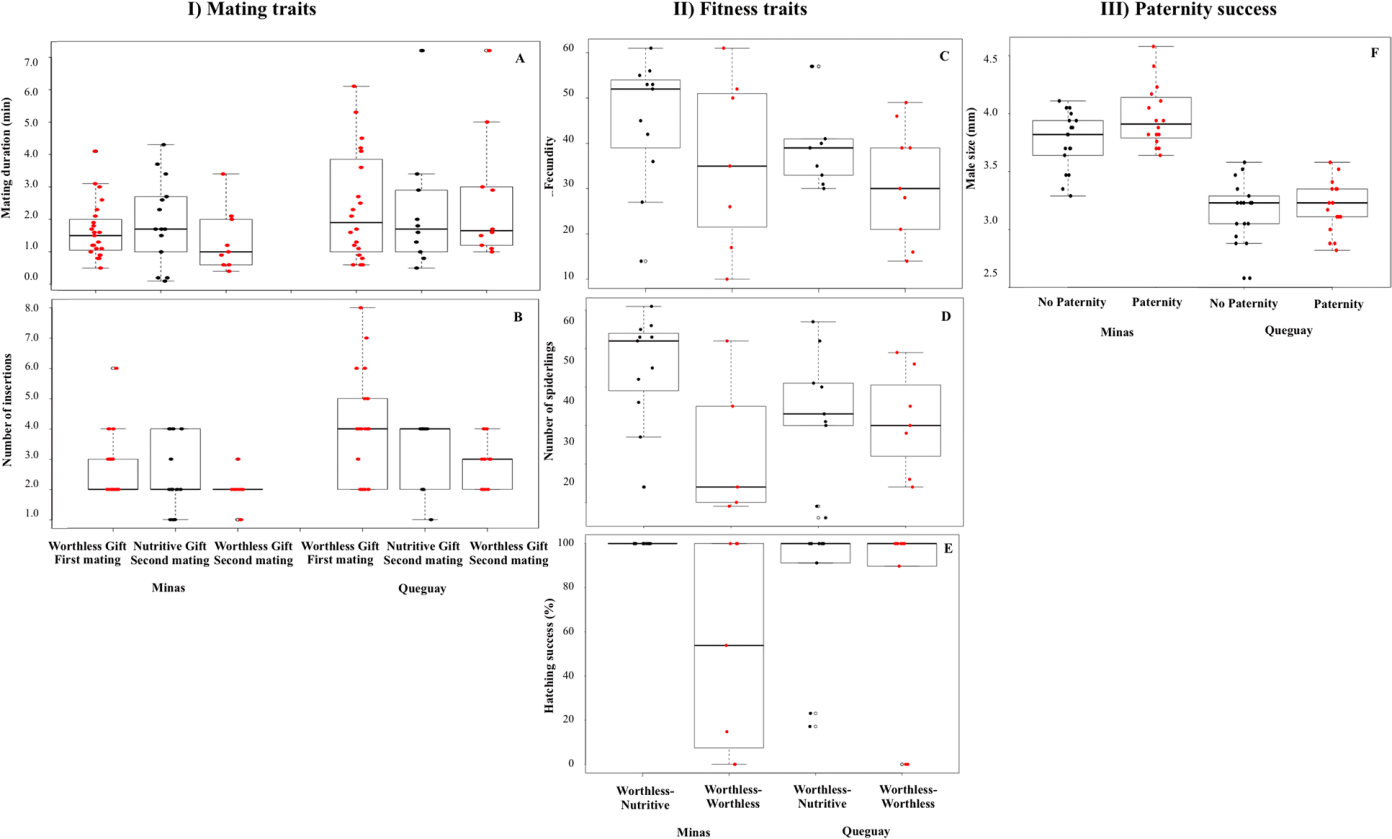
Mating, fecundity and paternity success of males offering worthless gifts. Double matings performed in a window period of 24 h in the two studied *Paratrechalea ornata* populations (Minas and Queguay). All females’ first matings involved males offering a worthless gift (*n*_Minas_ = 23, *n*_Queguay_ = 20), whereas second matings were either with males offering a nutritive (*n*_Minas_ = 14, *n*_Queguay_ = 10) or a worthless gift (*n*_Minas_ = 9, *n*_Queguay_ = 10). (I) Effects of gift type and mating order on mating duration (**A**) and number of pedipalp insertions (**B**). (II) Effects of group (worthless-nutritive, worthless-worthless) on fecundity (**C**), number of spiderlings (**D**), and hatching success (**E**). (III) Effects of male size on paternity success (**F**). In the boxplot, the black line represents the median; gray dots represent the outliers. In (I), black dots represent the data points of nutritive and red dots of worthless gifts; in (II), black dots the represent data points of the worthless-nutritive group and red dots of the worthless-worthless group; in (III), black dots represent the data points of zero paternity and red dots of paternity success. The detailed data information can be found at the “Availability of data and materials” section

We found no effect of gift type or mating order on *mating traits*, but there was a population effect, as males from Queguay performed more pedipalp insertions during mating than those from Minas (Table 3, Fig. 2AB). The models for the *fitness traits* indicated differences between groups and populations (Table 3, Fig. 2CDE). Females that received worthless gifts in the two matings had lower fecundity and fewer offspring with lower hatching success than those receiving a worthless and a nutritive gift. The significant interaction between group and population indicates that these effects were only found in Minas, as females from Queguay had similar fitness in both worthless-nutritive and worthless-worthless groups.

**Table 3:** Mating, fecundity, and paternity success of males offering worthless gifts. **(**I) Results from the GLMMs examining effects on *mating traits*: mating duration (min) and number of pedipalp insertions in relation to gift type (nutritive or worthless), mating order (first or second), population (Minas or Queguay), and female size. Initial models considered the interaction between gift type and populations and mating order and population. The model included female ID as random effect. (II) Results from GLM examining effects on *fitness traits*: latency of oviposition (days), fecundity (total number of eggs), number of spiderlings (hatched eggs), and hatching success (hatched eggs/total number of eggs) in relation to group (worthless-nutritive or worthless-worthless) in interaction with population (Minas or Queguay) and female size. (III) Results from the GLMM with binomial distribution examining the effects on paternity success (1/0) in relation to gift type (nutritive or worthless), mating order (first or second), population (Minas or Queguay), and male and female size (scale). The initial model considered as fixed effects: gift type, mating order, male and female size and sex sizes interaction, all terms in interaction with population, and included female ID as random effect. Significant *p* values are shown in bold

I. Mating traits	Mating duration				Number of insertions									
	Estimate	SE		P	Estimate	SE		P						
Gift type	0.03	0.10		0.76	− 0.24	0.20		0.23						
Mating order	− 0.03	0.08		0.70	− 0.32	0.18		0.07						
Population	− 0.15	0.13		0.27	0.49	0.19		**0.01**						
Female size	− 0.001	0.15		0.99	0.17	0.22		0.42						
	Random effects													
	Variance	SD				Variance	SD							
Female ID	0.02	0.14				0	0							
II. Fitness traits	Latency of oviposition			Fecundity					Number of spiderlings			Hatching success		
	Estimate	SE	P	Estimate	SE	P			Estimate	SE	P	Estimate	SE	P
Group	− 0.01	0.02	0.54	− 0.10	0.08	**0.001**			− 0.51	0.11	<** 0.0001**	− 0.46	0.14	0.08
Population	− 0.02	0.03	0.67	− 0.23	0.09	**0.001**			− 0.64	0.11	<** 0.0001**	− 0.46	0.14	**0.01**
Group*Population	0.01	0.03	0.70	− 0.19	0.12	0.10			0.37	0.14	**0.01**	0.56	0.19	**0.003**
Female size	− 0.02	0.02	0.40	− 0.21	0.09	0.06			− 0.53	0.13	0.56	− 0.42	0.16	0.11
III. Paternity success	Paternity													
	Estimate	SE		P										
Intercept	− 1.72	1.21		0.15										
Gift type	1.43	1.04		0.17										
Mating order	− 0.01	0.68		0.99										
Population	2.69	1.52		0.07										
Male size	0.79	0.54		0.14										
Female size	− 0.94	0.69		0.17										
Male size × female size	1.16	0.56		**0.04**										
Population × female size	1.59	1.27		0.21										
Population × gift type	− 2.16	1.28		0.09										
	Random effects													
	Variance	SD												
Female ID	0	0

#### Paternity success

The hypervariable STR locus D4 used for paternity analyses always detected the presence of maternal alleles and the alleles from one of the males; therefore, one male was the father of the whole clutch, and we did not observe mixed paternity clutches (e.g., Additional file: Fig. S2). In all cases, we could exclude from paternity the male whose alleles did not appear in the litter. In both populations, we found no effect of gift type or mating order on this outcome (Table 3). However, we found a significant interaction between male and female sizes. We explored this effect separately in each population and found that large males more often acquired the paternity of the clutch in Minas (*p* = 0.04) but not in Queguay (*p* = 0.69) (Fig. 2F).

## Discussion

The invasion and spread of new phenotypes have been investigated for irreversible and non-flexible strategies, in which different individuals expressing discrete alternative phenotypes can coexist, with these phenotypes usually maintained by negative frequency or density-dependent selection [2, 3, 6, 14]. In contrast, due to their low fitness success, deceptive phenotypes like worthless nuptial gifts are expected to be maintained in low frequencies in populations [10, 12–14, 39]. Here, we showed that male spiders performing the alternative tactic (worthless gifts) gain similar or higher fitness success than when using the original dominant one (nutritive gifts) under highly stressful environmental conditions. These findings are the first empirical evidence that a deceptive tactic can become a dominant tactic in a population, even when occurring in high frequency.

Evolutionary shifts toward optimal values in sexual traits in response to prevailing environmental conditions can favor the emergence and evolution of alternative mating tactics, which may in turn differ in their fitness consequences among populations [11, 40–42]. By studying two populations of the spider *P. ornata*, we show that males have different propensities for nuptial gift-giving tactics (nutritive versus worthless), whereas females have moved their pre- and post-copulatory choice away from gift content and toward assessing other male attributes (i.e., male size). The co-existence of nutritive and worthless gifts is possible in moderately stressful environments, with occasional prey limitations (Minas). But, when the environment becomes permanently highly stressful (Queguay), males almost completely replace the production of nutritive with worthless gifts. A recent study using six *P. ornata* populations diverging in their environmental stress levels verified these results, as worthless gifts are maintained in low proportions when the environment is less stressful and females penalize the mating duration of males offering these deceptive gifts [43].

**Fig. 3:**
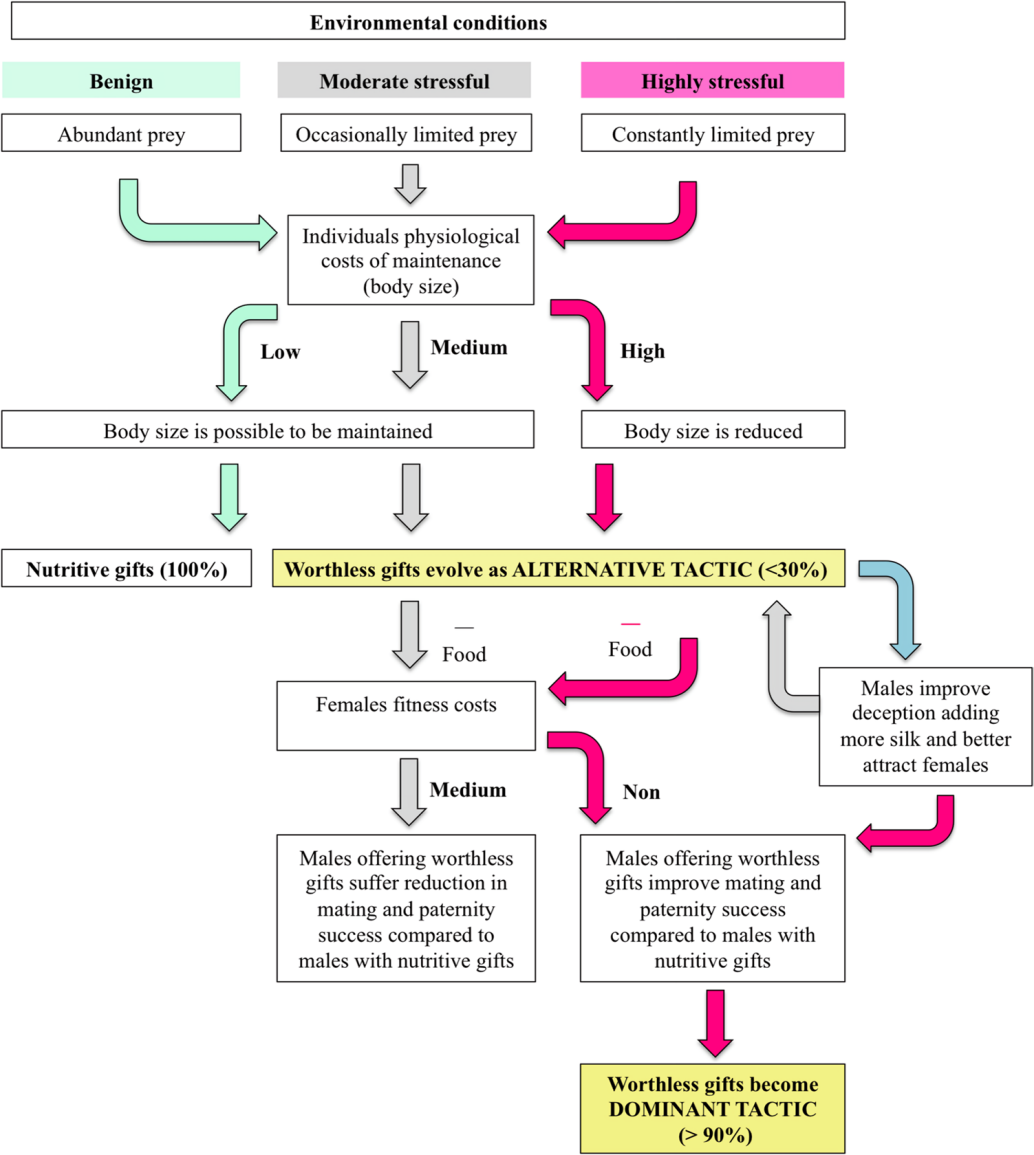
Theoretical outline proposing potential pathways for the evolution and spread of worthless nuptial gifts in spider populations under benign, moderate, and highly stressful environmental conditions. (I) In a scenario of benign conditions, prey are abundant and individuals have relatively low costs of body maintenance, and survival, hence, nutritive nuptial gifts are expected to be the dominant tactic adopted by males. (II) Under moderate stressful conditions, prey becomes occasionally limited, and the cost of individuals’ body maintenance and survival becomes relatively high. Males would reduce costs of gift production by offering worthless gifts as an alternative mating tactic. Males eventually improve deception by adding more silk, which increases female attraction. By receiving worthless gifts, large females would suffer costs and a reduction in fitness success; hence, they are expected to favor paternity of males offering nutritive gifts over those with worthless gift. Worthless gifts would be maintained in low percentage in the population. (III) Under highly stressful conditions, prey becomes constantly limited, and the cost of individuals’ body maintenance and survival becomes extremely high. Selection would favor small sizes in the population, maximizing individual fitness in the environment. Males would reduce costs of gift production by offering worthless gifts as an alternative mating tactic. Because of size reduction, small females would not suffer fitness costs when receiving worthless gifts, and hence, they are expected to favor paternity of males regardless of gift content. Worthless gifts would be favored, spread, and be maintained in high percentages in the population

According to the environmental threshold model, nuptial gifts can be considered a threshold trait that is environmentally sensitive [15, 16], but also has a heritable component [44–46]. At the individual level, this means that the probability of producing a nutritive or worthless gift varies among individuals [24]. Each male should have its own genetic switch point to trigger the production of different gift types, based on a threshold level of an environmental cue value (i.e., prey availability, or even the male’s condition) (this study, 23, 24). At the population level, this means that the frequency of mating tactics in each population will ultimately be determined by the local environmental conditions, and it will also be influenced by the frequency of alleles determining individual switch points. Thus, these genetic switch points are subject to changes under selection over evolutionary time. It is known that stressful conditions result in costs for the production and maintenance of sexual traits and can reduce the reproductive output of individuals in a population [47, 48]. As a result, males diminish costs associated with gift production by offering worthless gifts (Fig. 3). Overall, in the field, we found that the number of prey negatively affected the likelihood of offering a worthless gift. By eating the prey and wrapping leftovers in silk, small males can obtain energy and additionally have chances to continue courting and mating throughout the reproductive season. Producing a nutritive nuptial gift is predicted to be costly for males inhabiting the highly stressful environment of the Queguay population, and therefore, this context favors the maintenance of a very high percentage of worthless gifts (almost 100%). The fact that even under benign conditions, and in the presence of ample prey, males mostly produce worthless gifts (84–100%) in this population indicates that individuals from Queguay have a narrower plasticity in this behavioral trait than those from Minas (0–95%).

We propose that the potential for the worthless gifts to spread and fully invade is promoted by highly stressful environmental conditions and may involve at least two prerequisites: permanent prey limitation and a concomitant reduction in individual size (Fig. 3). In our study, this probably occurred due to idiosyncrasies of the regional climatic variation [49] and the riparian habitat. Local climate variation, in particular local precipitation, has been described as a relevant factor affecting variation in natural selection [50]. Individuals from *P. ornata* species live at freshwater courses, which are known to be highly unpredictable and stressful for various organisms [51, 52]. Under these natural conditions, the emergence of insect prey from the watercourses may vary strongly over time [53, 54] and can be markedly affected by floods [52]. This was corroborated in our populations by the large variation in prey number found during the sampling dates, and particularly the insect blooms associated with Minas but not with Queguay. As prey becomes constantly limited, the costs associated with individuals’ body maintenance and survival become high. Our results suggest that the number of prey *per capita* can be about six times higher in Minas than in Queguay. Therefore, in Queguay males are under much stronger selective pressure to secure enough resources for survival, while they also need to allocate prey to produce nuptial gifts compared to Minas. Prey limitation seems to permanently constrain individuals’ morphological traits in Queguay, and as a consequence selection favors small body size, minimizing maintenance costs and optimizing fitness under these particular environmental conditions [55–59]. While it is true that our experiment did not fully eliminate environmental or maternal effects on individuals because we collected them as juveniles, field data from 2011 [23] and 2017 (MJAlbo unpublished data) generated similar results.

The effects of stressful environments on life history traits can have important consequences for the co-evolution of female preferences [60–66]. For instance, in stickleback fishes, water eutrophication reduces male courtship effort and changes female preferences [48]. Here, we verified results from a previous study [67] demonstrating that *P. ornata* females from the moderate stressful environment (Minas) suffer fitness costs from mating with males offering worthless gifts (i.e., reduction in fecundity, number of spiderlings, and hatching success). In contrast, this did not happen to females from the highly stressful environment (Queguay). We explain these results based on the existing individual size differences between populations. Like males, large females from Minas would require more nutrients for their body maintenance and survival than small females from Queguay, and thus, food supply via the gift generates a divergent outcome in their fitness success (Fig. 3). Selective pressures for nutritive gifts are therefore highest for females in Minas. These conditions drive the maintenance of nutritive gifts in the population and can select a mating niche for males saving on costly courtship traits [68]. However, because a high percentage of males still offer worthless gifts, females choose cryptically, biasing paternity toward large males. Not surprisingly, large and high condition males in Minas (*high prey availability*) produced mostly worthless gifts, instead of nutritive ones. In this population, male size positively correlates with mating duration [38], indicating that male size and condition are the most important attributes that maximize their fitness, at least when mate competition is low. In contrast, as expected, females bias paternity toward one male, independently of gift content and male size, in Queguay. Which males’ attributes are under selection in this highly stressful population remains unknown, but sexual selection for gift content clearly plays a weaker role under unpredictable environmental conditions [47, 66]. Further research on the potential costs of nutritive and worthless gift production would be relevant to gain insights into the maintenance of both gift-giving tactics, specially due to worthless gift contain much more silk [24] and might provide additional benefits to both males and females [69].

## Conclusions

This study illustrates how deceptive and reversible phenotypes can diverge according to prevailing environmental conditions, revealing gift-giving spiders as a promising eco-evolutionary model [5, 33]. The balance between sexual selection (produce nutritive gifts) and natural selection (eat and survive) is expected to be disrupted in habitats with high unpredictable conditions [47]. Selection for gift content would eventually be limited in populations under highly stressful conditions, allowing worthless gifts to spread quickly and become the dominant tactic. In such a case, selective pressures for gift content are reduced, supporting the hypothesis that sexual selection becomes relaxed in harsh environments [47, 48, 70].

## Methods

### Biological model, sites, and samplings

*Paratrechalea ornata* is a species living in riparian habitats associated with watercourses in South America [71]. Individuals have crepuscular/nocturnal habits [72]. In Uruguay, the species has two reproductive periods, March to June and September to December, when males are observed carrying nuptial gifts and courting females along the stream edge [23].

All samplings were done at two localities in Uruguay: Minas, Lavalleja (34.278 S, 55.233 W) and Queguay, Paysandú (32.169 S, 57.477 W) during 2015–2020, referred to hereafter as Minas and Queguay, respectively. Both sites differ in basin grades and climatic conditions (temperature and precipitation) [49]. Uruguay is influenced by El Niño-Southern Oscillations (ENSO) [73, 74] that produces climatic heterogeneity, including substantial variation in rainfall [75]. El Niño influences on rainfall are stronger and create greater variation in the northern (Queguay population) region than in the southern (Minas population) one [49]. Thus, local climatic variations add unpredictability to the environment (e.g., regional variation in flood events). Consequently, in terms of climatic variation, the environmental conditions are moderate for organisms living in Minas, but highly stressful for those in Queguay.

### Statistical analyses

We analyzed the data using R version 4.1.1 [76], fitting generalized linear models (GLMs) and generalized linear mixed models (GLMMs) [77]. When performing model selection, we assessed competing models based on their Akaike Information Criterion (AIC), using ΔAIC > 2 as the threshold for considering a model significantly more parsimonious than an alternative one [78, 79]. All selected models were validated via residual analyses [77]. Detailed information and additional data analyses are described in the following sections. An overall scheme of the experimental approaches can be found in the Additional file: Fig. S1.

### Worthless gifts in natural populations: ecological and individual effects

We conducted the field study during 2015–2016 and measured the frequency of deceptive worthless gifts in the two natural populations. We collected individuals monthly during the second reproductive season (spring), from September to December in 2015 and 2016, totalling 16 dates (*N* = 8 in each population). Due to climatic conditions, the number of final sampling dates for statistical analyses was reduced (*N* = 15).

On each collection date, four persons manually sampled all spiders from the same patch for 1 h. The sampling was performed during the night (approximately 2200–2300 h) when the spiders were active. On each collection date, we also obtained samples of small invertebrates (a total of 23,732 potential spider prey) in the riparian habitat. We used a light trap consisting of a white fabric sheet (60 × 80 cm) and a camping lantern (10.5 cm diameter and 19.5 cm height) placed next to the water for 2 h (including the hour during which spiders were collected). All sampled invertebrates were preserved in 75% ethanol for later counting the number of individuals under a stereomicroscope. Upon collecting spiders, we recorded the number of adult males (with and without gifts) and females. In total, we collected 1163 spiders: 339 were adult females (*n* = 145 Minas; *n* = 194 Queguay), and 455 were adult males (*n* = 235 Minas; *n* = 220 Queguay), of which 151 were males carrying a nuptial gift (*n* = 99 Minas; *n* = 52 Queguay), accounting for 36% of males in Minas and 25% in Queguay, with no significant differences between populations (Additional file: Table S1). We also weighed (live body mass (g)) and measured the size (cephalothorax width (mm)) of each adult male and female, after which all animals were released at the site of collection. For adult males carrying a nuptial gift, we immediately removed the gift from the males’ chelicerae using tweezers and placed it in an Eppendorf tube. We weighed the gifts, dissected them using tweezers under a stereomicroscope, and recorded their contents. We classified the gifts according to a previous study [18] as “nutritive” (containing fresh prey) or “worthless” (containing prey leftovers, plant parts, or other non-nutritive items).

We used these data to estimate the following. (1) Reproductive variables: *proportion of males carrying a gift*—the number of males with a nuptial gift divided by the total number of adult males; *proportion of males carrying a worthless gift*—the number of males with a worthless gift divided by the number of males with a gift; and *Gift weight*, measured as gift mass (g). (2) Ecological variables: *Prey—*the total number of insect prey—and *prey per spider*, calculated as the total prey number divided by the total number of individual spiders. (3) Individual (male and female) measurements: *size*, measured as the cephalothorax width (mm), and *weight*, measured as body mass (g).

We examined the differences between populations (with population as the single independent variable) separately for each dependent variable using GLMs. We explored the proportion of males carrying a gift and the proportion of males having worthless gifts using GLMs with binomial distributions. We examined the gift weight and both males’ and females’ size and weight using GLMs with Gaussian distributions, and the number of prey and prey per spider (calculated as the total number of prey in relation to the total number of spiders each date) using GLMs with Poisson distributions. We log-transformed gift weight and prey number to meet assumptions of normality and homoscedasticity. In the next stage of the analyses, we used GLMMs with binomial distributions to assess the effect of the different independent variables (all variables scaled and not correlated) on the proportion of males carrying a worthless gift (reproductive variable). We based our inferences on a model selection approach [78], using the bias-corrected version of the Akaike Information Criterion (AIC_*c*_). Our set of candidate models included 22 models with the proportion of worthless gifts as the response variable, and every possible combination between the fixed effects of prey number (ecological variable), male and female size (individual variables), and population (“Pop” = Minas, Queguay), as well as single two-way interactions between each pair of these variables, as fixed effects. All models included the dates generated from a combination of month and year (2015 and 2016) as random effects. Models were fit using functions of the packages *lme4* [80] and *AICcmodavg* [81]*.*

### Common garden experiment: high and low prey availability

In 2020, we developed a laboratory experiment measuring the effect of the environment on morphological and behavioral traits, to understand the degree of differentiation within and between populations. We initiated this experiment with a subset of 162 juveniles: Minas (*n* = 74) and Queguay (*n* = 88). We weighed individuals immediately after collection, and they averaged 0.07 g (± 0.003 SE) in Minas and 0.04 g (± 0.001 SE) in Queguay. Spiders were housed individually in transparent plastic containers (9 cm diameter and 7 cm height) with pebbles as substrate and water provided *ad libitum* in a cotton ball, at room temperate averaging 22.0 °C and under a natural photoperiod. During the first 3 weeks after collection, we equally fed all juveniles from both populations (week 1, no food; week 2, five fruit flies (*Drosophila* sp.); week 3, ten fruit flies). This procedure allowed us to standardize the individual weights between and within populations. Because sex in spiders can only be determined by the genitalia after individuals reach adulthood [82], at this point, we could not assign sex to each individual. Thus, we randomly selected 89 individuals (43 in Minas and 46 in Queguay) and raised them under two different feeding treatments in each population for 12 weeks. *High* and *low prey availability* consisted of an alternated weekly feeding regimen of fruit flies during 8 weeks and house flies during 4 weeks. In the *high prey availability* regimen, spiders received a total of 168 fruit flies (21 per week) and 12 house flies (3 per week). In the *low prey availability*, spiders received a total of 56 fruit flies (7 per week) and 4 house flies (1 per week). To maximize the differences between the created experimental environments, the two feeding treatments also differed in prey accessibility over time: in the *high prey availability* treatment, individuals consistently received the food on the same days of the week (Monday, Wednesday, and Friday), while in the *low prey availability* treatment, the time between meals varied randomly (range: 2–11 days). The remaining spiders (*n* = 55; Minas = 25, Queguay = 30), which included the females used in the experiments, were fed like the males held under high prey availability. Once they reached 25 days after adulthood and were physiologically mature and receptive to reproduction [83], we exposed males to females. Couples were randomly paired. The developmental time of females averaged 44.1 days (± 0.75 SE), whereas that of males averaged 48.8 days (± 0.74 SE).

We examined how prey availability and population of origin interact and influence male worthless gift production by analyzing the behavioral responses from the four experimental male groups, created by the combination of the feeding treatment (*high* and *low prey availability*) and population (Minas and Queguay). The experimental groups were as follows: “High-Minas,” consisting of males from Minas reared under abundant prey availability (*n* = 22); “Low-Minas,” consisting of males from Minas reared under limited prey availability (*n* = 15); “High-Queguay,” consisting of males from Queguay reared under abundant prey availability (*n* = 25); and “Low-Queguay,” consisting of males from Queguay reared under limited prey availability (*n* = 19). In each experimental group, we allowed males to court females giving them equal opportunities for the gifts they could produce (i.e., wrap in silk), as they had access to a live housefly (*Musca domestica*) and to the exuviae of a mealworm, which males could thus use to make a nutritive or a worthless gift, respectively. A third option was also possible in this experiment, as males could produce a worthless gift after eating the housefly. Thus, we used two estimates to determine whether the housefly represented a nutritive or a worthless gift. First, we calculated the housefly consumption by males before wrapping it in silk as the difference between the final gift weight and the mean weight of 43 houseflies (mean ± SE: 0.0158 ± 0.0007 g). This resulted in the first classification in which worthless gifts represent between 78–98% of the housefly consumed (mean ± SE: 0.0019 ± 0.0004 g), whereas nutritive gifts included between 22 and 77% of the housefly consumed (mean ± SE: 0.0103 ± 0.0055 g). Second, we calculated the weight difference between housefly and natural gifts found in the field (mean ± SE: nutritive gift = 0.0046 ± 0.0003 g, worthless gift = 0.0014 ± 0.0001 g in Minas; nutritive gift = 0.0015 ± 0.0001 g, worthless gift = 0.0009 ± 0.0002 g in Queguay). This results in a 2.3 and 1.4 times difference for nutritive and worthless gifts in Minas and 6.8 and 2.1 times for nutritive and worthless gifts in Queguay. We transformed the housefly gift weights according to this scale, which allows for contrasting it with the field data.

We performed the experiments in transparent glass cages (30 × 14 cm base, 20 cm height), in which we simulated natural conditions by covering the bottom with pebbles and water [23, 34]. We placed a female in the experimental cage 24 h before introducing a male, allowing them to deposit silk that stimulates male courtship and gift production [84]. On the day of the experiment, we introduced the male together with six mealworm exuviae distributed along the bottom of the cage. Once the male started to court (fast leg and pedipalp vibrations), we offered him a live housefly with tweezers. By detecting the wing vibrations, males could capture the housefly [22, 23, 34, 35, 84] or proceed without it [24]. We repeated the action of offering the housefly every 15 min, until the male grabbed and caught it, or grabbed any mealworm exuviae from the pebbles. In all cases, we finished the trial when the male wrapped an item in silk and offered it to the female or 4 h after the start of the trial. If, at the end of the 4 h, the male grabbed an item but did not wrap it in silk, we left him and checked for a wrapped gift the next day. In the cases where the males did not grab any item, we finished the trial after 4 h and repeated the experiment for the male a week later (this was done up to 3 times). In all cases, we prevented males from mating using a paintbrush. We recorded which item (housefly or exuviae) the male grabbed and wrapped in silk during each trial. We recorded the latency and duration of silk wrapping, as well as the number of silk wrapping bouts. We calculated the total silk wrapping duration (min) as the sum of all wrapping bouts durations. We also recorded the latency of gift offering as the time the male finished the gift wrapping until he offered it to the female, and the occurrence of female acceptance, determined on the basis as to whether she grabbed the gift.

We examined males’ and females’ size (mm) and weight (g) by performing a GLM with Gaussian distribution in a fully factorial model including feeding treatment, population, and their interaction. Additionally, we performed GLMs for the different response variables concerning gift type (nutritive: 0, worthless: 1), population (Minas, Queguay), feeding treatment (high, low prey availability), and the interaction between population and feeding treatment. These GLMs used Gaussian error distributions for the gift weights, silk wrapping duration and latency of gift offering, Poisson error distributions for the number of silk wrapping bouts, and Binomial error distributions for female acceptance (accept: 1, no accept: 0).

### Mating, fecundity, and paternity success of males offering worthless gifts

In 2020, we explored the fitness effects and paternity success associated to male gift type (nutritive or worthless). We used a subset of 129 juveniles from Minas (*n* = 69) and Queguay (*n* = 60). Spiders were maintained under the same conditions described for the previous experimental setup and fed under high prey availability; individuals’ weights and sizes and statistical analyses are given in Additional file 1: Table S4.

Previous data suggest no effect of mating order on fecundity (total number of eggs) in *P. ornata* [38]. Nevertheless, we controlled for mating order and gift type by creating two double mating groups in which females always received a worthless gift in their first mating, then received either a nutritive or worthless gift in the second mating. We exposed females sequentially to mate with two different males offering wrapped gifts in a time window of 24 h. In the worthless-nutritive group (Minas, *n* = 14; Queguay, *n* = 10), the female mated first with a male offering a worthless gift consisting of an exuviae of a mealworm, and second, with a male offering a nutritive gift containing a recently caught housefly. In the worthless-worthless group (Minas, *n* = 9; Queguay, *n* = 10), the female mated twice with males offering a worthless gift (exuviae). Couples were paired according to their unique locus D4 (see below), but both mating order and gift type were randomly assigned to males.

#### Mating and fecundity traits

We performed the experiments under the same conditions as the previous experimental setup (common garden). The female was placed in the arena alone for 24 h; on the day of the experiment, we enclosed her with a glass container and placed the male in the cage. Once he sensed the female silk and started to court, we offered him a housefly for producing a nutritive gift. Once the male caught the housefly, we gave him 20 min to wrap it in silk; this procedure assured all males offered a nutritive gift. For the worthless gifts, we deposited six mealworm exuviae along with the pebbles, so the male could wrap one in silk. After the male finished the silk wrapping of the gift and started walking, vibrating legs, and searching for the female, we released the female and allowed the male to offer the gift and mate. We registered the number and duration of pedipalp insertions (male copulatory organs) and calculated the total mating duration (a proxy of sperm transfer) as the sum of the durations of all insertions (min). The experiment finished when the couple separated and the female departed with the gift.

After the two matings were completed, we fed females with a mixed feeding regime of fruit flies and houseflies until they constructed an egg sac and spiderlings had emerged. We recorded the construction date of the egg sac, calculating the latency to oviposition as the period from the date of the second mating to the date of egg sac construction. Some females did not produce an egg sac; thus, the sample sizes for the fitness traits for each population were: Minas (worthless-nutritive = 11, worthless-worthless = 7) and Queguay (worthless-nutritive = 9, worthless-worthless = 9). Once spiderlings emerged, we counted the hatched and unhatched eggs and calculated female fecundity as the sum of both. Additionally, we computed spiderlings hatching success as the proportion of hatched spiderlings over the fecundity. We preserved the spiderlings in ethanol 95% once they hatched.

We first used GLMMs with gamma and Poisson distributions for modeling the *mating traits*: mating duration and number of insertions, respectively, including gift type (nutritive or worthless), mating order (first or second), population (Minas or Queguay), and female size as fixed effects, and female ID as a random effect. Second, we used GLMs to examine the *fitness traits*: latency of oviposition, fecundity, number of spiderlings, and hatching success using group (Worthless-nutritive or worthless-worthless) in interaction with population (Minas or Queguay) and female size as fixed effects. We used Poisson error distributions for fecundity and number of spiderlings, and Binomial error distributions for hatching success.

#### Paternity success

For the paternity assessment, we used the hypervariable STR (Short tandem repeat) locus D4, isolated and reported for a species of the genus *Paratrechalea* [85] and optimized for *P. ornata* [86]. We genotyped individuals using DNA extractions from the adulthood molt, which allowed us to identify each individual according to this single locus. Before the mating experiments, we assembled the triads (a female and two males) with at least one exclusive allele (not shared between them or with the female). We genotyped and estimated the proportion of paternity based on the entire clutch of spiderlings from each female, using the competitive microsatellite PCR technique [87, 88]. This technique allows us to establish each male’s relative contribution to paternity by examining the presence of each exclusive allele. We additionally verified the technique performance and performed a positive control by genotyping equimolar mixtures of DNA extractions from individuals with unique alleles.

We extracted total DNA using a standard protocol (modified from [89]) and quantified DNA concentrations from extractions using nanodrop (NANODROP LITE Spectrophotometer, Thermo Scientific). We carried out the amplification by PCR following [86]. Successful amplifications were analyzed by electrophoresis on an ABI 3130 Genetic Analyser housed in the Unidad de Biología Molecular of the Institut Pasteur (Montevideo, Uruguay). A LIZ600 size standard was included in all lanes. The fragment sizes were determined, and genotypes were assigned using the Peak Scanner Software v1 software (Applied Biosystems).

We examined *paternity success* (1/0) with a GLMM with binomial error distributions, including gift type (nutritive or worthless), mating order (first or second), population (Minas or Queguay), and male and female size as fixed effects, and female ID as a random effect. All initial models incorporated the interactions among fixed effects, which were subsequently removed, whenever they were not found to have significant effects on the model fit. 

## Supplementary Information

**Additional file 1: Fig. S1.** Scheme of experimental approaches. We used three approaches: 1) Field study, we explored the frequencies of worthless nuptial gifts, prey availability and individuals’ size and weight in two natural populations with moderate and highly stressful conditions. 2) Laboratory experiment 1, we developed a common garden experiment raising individuals from both populations under high and low prey availability and recording the frequencies of worthless nuptial gifts and individuals size and weight. 3) Laboratory experiment 2, we performed double mating experiments exposing females to males offering nutritive or worthless gifts in both populations and examined mating, fitness and paternity success. **Fig. S2.** Example of paternity visualization. Graphical view from PickScanner program used for paternity exclusion. Each pick represents a different allele. 1) Male 1 with alleles sized 214 and 218bp; 2) Male 2 with alleles sized 200 and 214; 3) Female with alleles sized 206 and 210bp; 4) offspring with alleles sized 200, 206, 210 and 214bp. Exclusive alleles from male 1 (sized 218bp) is not in the offspring, so this male can be excluded from paternity.

## Data Availability

All data generated and analyzed during this study are included in this published article and its supplementary information files and publicly available at the Mendeley repository [90].
