# Supplementary material for: Stressful environments favor deceptive alternative mating tactics to become dominant
Source: BMC Biol. 2023 Jul 27;21:162. doi: 10.1186/s12915-023-01664-5 (PMC10375696; doi:10.1186/s12915-023-01664-5)
Supplement: Supplementary file 1 — Additional file 1: Fig. S1. Scheme of experimental approaches. We used three approaches: 1) Field study, we explored the frequencies of worthless nuptial gifts, prey availability and individuals’ size and weight in two natural populations with moderate and highly stressful conditions. 2) Laboratory experiment 1, we developed a common garden experiment raising individuals from both populations under high and low prey availability and recording the frequencies of worthless nuptial gifts and individuals size and weight. 3) Laboratory experiment 2, we performed double mating experiments exposing females to males offering nutritive or worthless gifts in both populations and examined mating, fitness and paternity success. Fig. S2. Example of paternity visualization. Graphical view from PickScanner program used for paternity exclusion. Each pick represents a different allele. 1) Male 1 with alleles sized 214 and 218bp; 2) Male 2 with alleles sized 200 and 214; 3) Female with alleles sized 206 and 210bp; 4) offspring with alleles sized 200, 206, 210 and 214bp. Exclusive alleles from male 1 (sized 218bp) is not in the offspring, so this male can be excluded from paternity. [file 12915_2023_1664_MOESM1_ESM.docx]

**Additional File**

**Albo et al 2023. Stressful environments favor deceptive alternative mating tactics to become dominant. *BMC Biology***

**TABLES - Additional File**

**Table S1. Worthless gifts in natural populations: ecological and individual effects.** Mean value, standard error (SE), range and statistical analyses (GLM) for the assessed variables in each population along the reproductive season: proportion of males with a gift (number of males carrying a gift/total number of males), proportion of males with a worthless gift (number of males carrying a worthless gift/total number of males carrying a gift), gift weight (mg), male and female size (mm), male and female weight (mg) prey number (total number of potential prey collected in the area), prey per spider (total number of available prey/total number of spiders). Sample sizes in Minas: dates = 8, observations = 224, in Queguay: dates = 7, observations= 164. Significant effects are highlighted in bold.

|  | **Minas** | | | **Queguay** | | | **Statistics** | | |  |
| --- | --- | --- | --- | --- | --- | --- | --- | --- | --- | --- |
|  | **N** | **Mean** ± **SE** | **Range** | **N** | **Mean** ± **SE** | **Range** | | **Estimate** | **SE** | **P** |
| Nuptial gifts proportion | 7 | 0.36 ± 0.072 | 0.07-0.82 | 7 | 0.25 ± 0.068 | 0.0-0.63 | | -0.06 | 0.39 | 0.87 |
| Worthless gifts proportion | 7 | 0.38 ± 0.035 | 0.0-0.80 | 5 | 0.96 ± 0.02 | 0.90-1.0 | | 3.18 | 0.62 | **<0.0001** |
| Gift Weight | 98 | 0.0033 ± 0.0005 | 0.0001-0.0126 | 52 | 0.0009 ± 0.0004 | 0.0001-0.0023 | | -1.19 | 0.14 | **<0.0001** |
| Female size | 128 | 4.45 ± 0.005 | 3.20-5.90 | 115 | 3.80 ± 0.005 | 3.00-4.90 | | -0.64 | 0.05 | **<0.0001** |
| Female weight | 128 | 0.158 ± 0.001 | 0.050-0.280 | 115 | 0.095 ± 0.001 | 0.040-0.200 | | -0.07 | 0.005 | **<0.0001** |
| Prey number | 8 | 2611 ± 8.45 | 3-10000 | 7 | 405.7 ± 2.91 | 7-1000 | | 0.07 | 0.23 | 0.74 |
| Prey per spider | 8 | 38.8 ± 1.04 | 0.03-169.5 | 7 | 6.13 ± 0.32 | 0.08-13.3 | | 0.01 | 0.03 | 0.57 |

**Table S2**. **Common garden experiment: high and low prey availability**. Results from the Generalized Lineal Models (GLMs) of males and females’ size (mm) and weight (mg) between populations, feeding treatment and the interaction between population and feeding treatment. Significant p values are shown in bold.

|  | **Population** | | | **Feeding treatment** | | | **Population*Feeding treatment** | | |
| --- | --- | --- | --- | --- | --- | --- | --- | --- | --- |
|  | **Estimate** | **SE** | **P** | **Estimate** | **SE** | **P** | **Estimate** | **SE** | **P** |
| Male size | -0.68 | 0.07 | **< 0.0001** | -0.16 | 0.08 | **0.04** | 0.21 | 0.10 | **0.04** |
| Male weight | -0.05 | 0.004 | **< 0.0001** | -0.03 | 0.01 | **< 0.0001** | 0.02 | 0.01 | **0.003** |
| Female size | -0.59 | 0.05 | **< 0.0001** | -0.02 | 0.07 | 0.68 | -0.04 | 0.09 | 0.68 |
| Female weight | -0.43 | 0.05 | **< 0.0001** | -0.03 | 0.06 | 0.56 | -0.01 | 0.08 | 0.82 |

**Table S3.** **Common garden experiment: high and low prey availability.** Mean and SE of silk wrapping duration (min), number of silk wrapping bouts (total number of silk wrappings), latency of gift offering (time from when males stop wrapping the gift until he offers to female in min) and female acceptance (frequency of females grabbing the gift) between populations (Minas or Queguay), feeding treatment (High or Low) and type of gift (nutritive or worthless). Asterisks indicate a change in the sample size due to missing values.

|  | **Minas** | | | | **Queguay** | | | |
| --- | --- | --- | --- | --- | --- | --- | --- | --- |
|  | **High** | | **Low** | | **High** | | **Low** | |
|  | **Nutritive**  (N =1) | **Worthless**  (N = 21) | **Nutritive**  (N = 6) | **Worthless**  (N = 9) | **Nutritive**  (N = 4) | **Worthless**  (N = 21) | **Nutritive**  (N = 2) | **Worthless**  (N = 17) |
| Silk wrapping duration | 1.0 ± 0 | 4.31 ± 0.48 | 1.08 ± 0.25  * | 3.41 ± 0.65  ** | 2.15 ± 0.41 | 5.02 ± 0.49 | 1.95 ± 0.54 | 3.10 ± 0.51  **** |
| Number of silk wrapping bouts | 1 ± 0 | 2.60 ± 0.24 | 1 ± 0  * | 2.25 ± 0.72  ** | 1 ± 0 | 2.52 ± 0.24 | 2.0 ± 0.99 | 2.0 ± 0.27  **** |
| Latency of gift offering | 11.5 ± 0 | 7.45 ± 1.61 | 18.38 ± 8.59  * | 6.78 ± 2.24  ** | 9.17 ± 6.32 | 9.58 ± 2.34  *** | 7.6 ± 0.29 | 23.97 ± 10.03  **** |
| Female acceptance | 1 ± 0 | 0.95 ± 0.05 | 0.83 ± 0.16 | 1 ± 0 | 1 ± 0 | 1 ± 0 | 0.50 ± 0.49 | 0.87 ± 0.09  **** |

*N = 5; **N = 8; ***N = 20; ****N = 15

**Table S4. Mating, fecundity and paternity success of males offering worthless gifts.** Mean value, standard error (SE), and statistical analyses (GLM) for male and female size (mm), male and female weight (mg). Significant effects are highlighted in bold.

|  |  | **Minas** | | **Queguay** | |  | | | | | **Statistics** | | | | | |
| --- | --- | --- | --- | --- | --- | --- | --- | --- | --- | --- | --- | --- | --- | --- | --- | --- |
|  |  | **Mean** ± **SE** |  | | **Mean** ± **SE** | **Group** | | | | **Population** | | | | **Group*Population** | | |
|  | **N** |  | **N** | |  | **Estimate** | | **SE** | **P** | **Estimate** | | **SE** | **P** | **Estimate** | **SE** | **P** |
| Male size (mm) | 41 | 3.86 ± 0.04 | 39 | | 3.17 ± 0.04 | 0.02 | 0.02 | | 0.35 | -0.19 | | 0.02 | **< 0.0001** | -0.01 | 0.03 | 0.83 |
| Male weight (mg) | 46 | 0.118 ± 0.001 | 40 | | 0.067 ± 0.002 | 0.04 | 0.05 | | 0.33 | -0.55 | | 0.05 | **< 0.0001** | -0.06 | 0.07 | 0.40 |
| Female size (mm) | 19 | 4.07 ± 0.08 | 19 | | 3.38 ± 0.06 | 0.01 | 0.02 | | 0.58 | -0.13 | | 0.03 | **< 0.0001** | -0.10 | 0.03 | **0.003** |
| Female weight (mg) | 23 | 0.124 ± 0.005 | 20 | | 0.084 ± 0.002 | 0.03 | 0.05 | | 0.53 | -0.31 | | 0.04 | **< 0.0001** | -0.12 | 0.07 | 0.11 |

**FIGURES- Additional File**


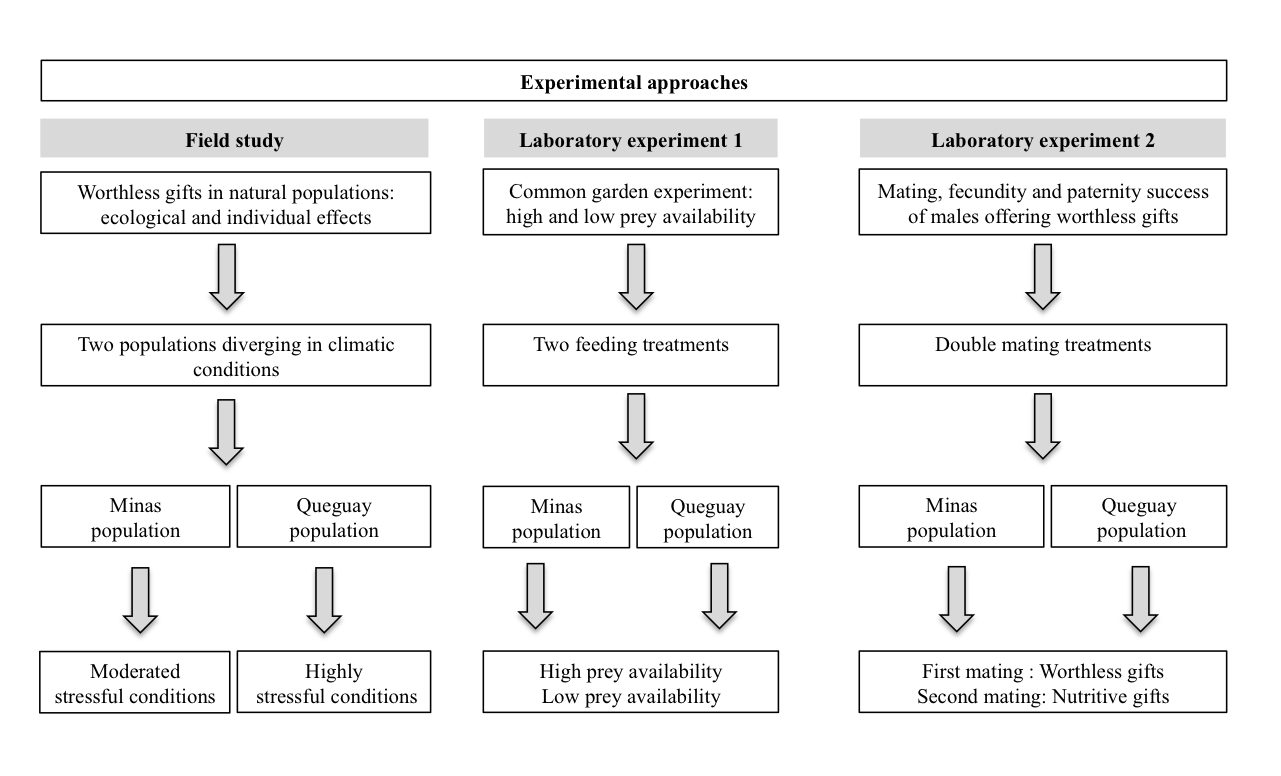
**Figure S1. Scheme of experimental approaches.** We used three approaches: 1) Field study, we explored the frequencies of worthless nuptial gifts, prey availability and individuals’ size and weight in two natural populations with moderate and highly stressful conditions. 2) Laboratory experiment 1, we developed a common garden experiment raising individuals from both populations under high and low prey availability and recording the frequencies of worthless nuptial gifts and individuals size and weight. 3) Laboratory experiment 2, we performed double mating experiments exposing females to males offering nutritive or worthless gifts in both populations and examined mating, fitness and paternity success.

**Figure S2. Example of paternity visualization**. Graphical view from PickScanner program used for paternity exclusion. Each pick represents a different allele. 1) Male 1 with alleles sized 214 and 218bp; 2) Male 2 with alleles sized 200 and 214; 3) Female with alleles sized 206 and 210bp; 4) offspring with alleles sized 200, 206, 210 and 214bp. Exclusive alleles from male 1 (sized 218bp) is not in the offspring, so this male can be excluded from paternity.
